# Identification and Expression Analysis of *EPSPS* and *BAR* Families in Cotton

**DOI:** 10.3390/plants12193366

**Published:** 2023-09-23

**Authors:** Zhao Li, Zhen Zhang, Yinbo Liu, Yuanqi Ma, Xing Lv, Dongmei Zhang, Qishen Gu, Huifeng Ke, Liqiang Wu, Guiyin Zhang, Zhiying Ma, Xingfen Wang, Zhengwen Sun

**Affiliations:** State Key Laboratory of North China Crop Improvement and Regulation, Key Laboratory for Crop Germplasm Resources of Hebei, College of Agronomy, Hebei Agricultural University, Baoding 071000, China; lizhao_4180@163.com (Z.L.); zhangzhen9808@163.com (Z.Z.); 17631706775@163.com (Y.L.); 15663010607@163.com (Y.M.); 15733256395@163.com (X.L.); zhangdongmei1108@163.com (D.Z.); guqishen0918@163.com (Q.G.); kehuifeng123@126.com (H.K.); wuliqiang@hebau.edu.cn (L.W.); mhyzh@hebau.edu.cn (G.Z.); mzhy@hebau.edu.cn (Z.M.)

**Keywords:** cotton, *EPSPS*, *BAR*, gene family, expression analysis

## Abstract

Weeds seriously affect the yield and quality of crops. Because manual weeding is time-consuming and laborious, the use of herbicides becomes an effective way to solve the harm caused by weeds in fields. Both 5-enolpyruvyl shikimate-3-phosphate synthetase (*EPSPS*) and acetyltransferase genes (bialaphos resistance, *BAR*) are widely used to improve crop resistance to herbicides. However, cotton, as the most important natural fiber crop, is not tolerant to herbicides in China, and the *EPSPS* and *BAR* family genes have not yet been characterized in cotton. Therefore, we explore the genes of these two families to provide candidate genes for the study of herbicide resistance mechanisms. In this study, 8, 8, 4, and 5 *EPSPS* genes and 6, 6, 5, and 5 *BAR* genes were identified in allotetraploid *Gossypium hirsutum* and *Gossypium barbadense*, diploid *Gossypium arboreum* and *Gossypium raimondii*, respectively. Members of the *EPSPS* and *BAR* families were classified into three subgroups based on the distribution of phylogenetic trees, conserved motifs, and gene structures. In addition, the promoter sequences of *EPSPS* and *BAR* family members included growth and development, stress, and hormone-related *cis*-elements. Based on the expression analysis, the family members showed tissue-specific expression and differed significantly in response to abiotic stresses. Finally, qRT-PCR analysis revealed that the expression levels of *GhEPSPS3*, *GhEPSPS4,* and *GhBAR1* were significantly upregulated after exogenous spraying of herbicides. Overall, we characterized the *EPSPS* and *BAR* gene families of cotton at the genome-wide level, which will provide a basis for further studying the functions of *EPSPS* and *BAR* genes during growth and development and herbicide stress.

## 1. Introduction

With the global climate and environmental change, the growth and development of crops are affected by various stresses, such as drought, salt, low temperatures, and weeds [1]. These stresses affect plants by changing plant physiological and metabolic reactions, causing irreversible damage or even death, and ultimately affecting crop yield [2]. Among them, weeds are an obstacle in the agricultural production process, which not only competes with crops for sunlight, water, and nutrients but also spreads some diseases and pests, which greatly threaten the growth of crops [3,4]. Therefore, weed control is a problem that cannot be ignored in agricultural production; it will affect the yield and quality of crops [5]. At present, the main method used to control weeds is to spray chemical herbicides. However, herbicides are limited because of their broad spectrum of extinction in agriculture, which also causes crops to be damaged while weeds are controlled in the field [6,7].

Currently, the most widely used herbicides are glyphosate and glufosinate [8]. For each of these herbicides, there are specific genes that can inhibit it. Firstly, 5-enolpyruvyl shikimate-3-phosphate synthetase (*EPSPS*) is the only enzyme that targets glyphosate, the most widely used broad-spectrum herbicide today [9]. The inhibition of glyphosate on *EPSPS* enzyme will increase the content of shikimic acid and hinder the synthesis of phenylalanine, tryptophan, and tyrosine of aromatic amino acids, thus causing the plant to wither and die [10,11]. Therefore, the expression level of *EPSPS* can directly affect the resistance of plants to glyphosate. Phenylalanine, tyrosine, and tryptophan are the essential amino acids of the three aromatic groups in plants, which need to be synthesized by the shikimic acid pathway [12]. However, *EPSPS* is a key enzyme in the shikimic acid pathway, which is widely found in higher plants and microorganisms. It can catalyze the production of EPSP synthetase from shikimic acid 3-phosphate (S3P) and phosphopyruvate (PEP) in chloroplasts, and eventually produce hormones and other important plant metabolites through this step, including growth kinins, aromatic amino acids, lignin, flavonoids, phenols, salicylic acid, and other secondary metabolites involved in plant defense [13,14]. As an important component of plant and microbial survival, the biosynthesis of aromatic amino acids and aromatic compounds through the shikimic acid pathway is essential for their continued existence [15]. The second is the acetyltransferase *BAR* gene cloned from the soil bacteria *Streptomyces absorbentus*, which is resistant to phosphinothricin (PPT), the active component of the herbicide PPT [16]. PPT is a potent inhibitor of glutamine synthase (GS), a key enzyme in the nitrogen assimilation pathway. When glutamine synthase is inhibited, NH_3_ accumulates, resulting in plant toxicity and death [16]. The herbicides with PPT activity have a conductive type of extermination, a wide range of herbicides, and can kill both above-ground and underground parts of plants [17]. *BAR* gene encodes phosphinothricin acetyltransferase (PAT), and PAT protein can free aminoacetylation of the active ingredient of herbicide PPT, thus detoxifying it so that it cannot inhibit the activity of GS [16,17]. The mechanism of resistance is the production of enzymes or enzyme systems that modify herbicides and degrade or detoxify them before they can act [18].

*EPSPS* has multiple homologous genes in plants and may play a key role in plant growth and development. Two *EPSPS* genes have been found in *Arabidopsis thaliana* and *Arabidopsis lyrata*, respectively [19], and three *EPSPS* homologous genes were found in wheat and rice, respectively [20,21]. Two *EPSPS* genes were found in tobacco and petunia, respectively [22]. In addition, glyphosate-resistant and glufosinate-resistant crops have been widely developed through transgenic. Transgenic *CP4 EPSPS* corn NK603 has a high tolerance to glyphosate [23]. Transgenic rice with the *CP4 EPSPS* gene can tolerate up to 1% of the commercial herbicide Roundup, which has a significant effect on overcoming the weed threat [24]. Transgenic tobacco with the *EPSPS* gene had a higher tolerance to herbicide stress [25]. In soybeans, co-expression of *G2*-*EPSPS* and *GAT* genes confers a high tolerance to the soybean herbicide glyphosate [26]. *The BAR* gene is widely used as herbicide-resistant gene in genetic engineering breeding, and it is also a marker gene in genetic transformation [27]. Transgenic *BAR* sweet potato can normally grow and develop under the stress of glufosinate basta [28]. Transgenic *BAR* wheat can tolerate very high concentrations of glufosinate and has no effect on yield [29]. The expression of the *BAR* gene was the highest in leaves of herbicide-tolerant maize with the *BAR* gene, which showed a high tolerance to glufosinate [30]. In addition, *EPSPS* and *BAR* have also been applied in cotton. Glyphosate-resistant cotton strain *pGR79 EPSPS*-*pGAT* showed a five-fold increase in resistance to glyphosate [31]. Transgenic *EPSPS*, *Cry1Ac,* and *Cry2Ab* three-gene cotton line NIBGE-E2 can tolerate herbicide at 1100 mL/Acre [32]. Transgenic *G2*-*aroA* cotton K312 has high resistance to glyphosate [33]. *BAR*-transgenic cotton BR001 is resistant to 20 mL·L^−1^ glufosinate herbicide [34,35].

Cotton is an important cash crop and is also the main natural fiber raw material of the textile industry [36]. With the completion of cotton genomes, diploid and tetraploid cotton genomes have been sequenced. Two highly homologous *EPSPS* genes were identified in *G. hirsutum* ‘Y18′ [37], and four *EPSPS* genes were found based on *G. hirsutum* ‘TM-1′ genome [38]. In addition, two *EPSPS* genes were discovered in *G. raimondii* [19], which are also highly homologous. However, there is no comprehensive identification among diploid and tetraploid cotton genomes. For the *BAR* genes, there is no report on the genome-level analysis of the *BAR* family gene in cotton. In recent years, the emergence of higher quality heterotetraploid genome sequences of *G. hirsutum* NDM8 and *G. barbadense* Pima90 has facilitated the systematic identification of this family of genes [39].

In this study, we will identify and analyze the *EPSPS* and *BAR* families of four cotton genomes through bioinformatics methods and tools and reveal the evolutionary mechanism of *EPSPS* and *BAR* genes. Further, the expression pattern of these genes was investigated under herbicide stress. The results will provide insights into the *EPSPS* and *BAR* gene families during growth and development and candidate genes for further study on the mechanism of herbicide resistance in cotton.

## 2. Results

### 2.1. Identification of EPSPS and BAR Family Gene Members

**Figure 1:**
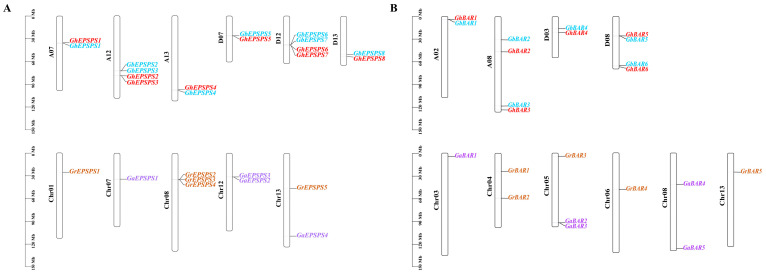
Chromosome distribution of *EPSPS* (**A**) and *BAR* (**B**) family members identified from four cotton species. Gh is red, Gb is blue, Ga is purple, and Gr is brown.

Based on the genomes of *G. hirsutum* NDM8, *G. barbadense* Pima90, *G. arboreum,* and *G. raimondii*, a total of 25 *EPSPS* gene sequences were detected in the four cotton species. In total, 8, 8, 4, and 5 *EPSPS* genes were identified, respectively (Table 1). They were named *GhEPSPS1*~*GhEPSPS8*, *GbEPSPS1*~*GbEPSPS8*, *GaEPSPS1*~*GaEPSPS4*, and *GrEPSPS1*~*GrEPSPS5* in order according to their distribution characteristics on the chromosomes (Figure 1A). The *EPSPS* family members identified in *G. hirsutum* and *G. barbadense* were distributed on chromosomes A07, A12, A13, D07, D12, and D13, with two *EPSPS* family members on both A12 and D12, which in *G. arboreum* were distributed on Chr07, Chr12 and Chr13, with two genes on Chr12. In *G. raimondii*, they were distributed on Chr01, Chr08, and Chr13, with three genes on Chr08. We found that *GhEPSPS* and *GbEPSPS* genes not only share the same number but also have similar gene structures, indicating that *EPSPS* genes are highly conserved between *G. hirsutum* and *G. barbadense*.

Similarly, a total of 22 *BAR* genes were detected in four cotton genomes. In all, 6, 6, 5, and 5 *BAR* family members were identified (Table 2). They were named *GhBAR1*~*GhBAR6*, *GbBAR1*~*GbBAR6*, *GaBAR1*~*GaBAR5*, and *GrBAR1*~*GrBAR5* in order according to their distribution characteristics on the chromosomes (Figure 1B). The *BAR* family members identified in both *G. hirsutum* and *G. barbadense* were distributed on chromosomes A02, A08, D03, and D08, with 2 *BAR* family members on both A08 and D08, which in *G. arboreum* were distributed on Chr03, Chr05 and Chr08, with 2 genes on Chr05 and Chr08. In *G. raimondii*, *BAR* genes were found on Chr04, Chr05, Chr06 and Chr13, with 2 genes on Chr04. Interestingly, the number of *BAR* family members, chromosome distribution, and structural similarities between the two tetraploid cotton species are also consistent, suggesting that *BAR* genes are highly conserved between *G. hirsutum* and *G. barbadense*.

### 2.2. Sequence Characterization and Protein Properties of EPSPS and BAR Family Members

Analysis of the properties of the four cotton *EPSPS* genes revealed that the full length of all family members was between 1797 and 4159 bp, except for the *GbEPSPS3* and *GaEPSPS3* (915 bp). The number of exons of *EPSPS* family members ranged from four to nine, with *GaEPSPS1* and *GrEPSPS2* having the most exons (nine) and *GbEPSPS3* and *GaEPSPS3* having only four exons. Analysis of the physicochemical properties of the proteins showed that the number of amino acids ranged from 185~521 aa, the molecular masses ranged from 20.56~55.54 kDa, and the theoretical isoelectric points ranged from 5.67~8.73 (Table 1).

Among the four cotton *BAR* family members, the full length of all family members ranged from 1627~5007 bp, except for the *GbBAR4* (14,746 bp) gene, which was the longest, and *GaBAR1* (561 bp), *GrBAR3* (957 bp), *GrBAR4* (508 bp) and *GrBAR5* (992 bp), which were shortest. The number of exons for the *BAR* family members ranged from one to six, with *GhBAR1*, *GhBAR6*, *GbBAR3*, *GbBAR6*, and *GrBAR2* having the most exons (6), while *GhBAR1*, *GaBAR1* and *GrBAR3* had only one exon (Table 2). The physicochemical properties of the proteins were analyzed, with amino acid numbers ranging from 89~277 aa, molecular masses between 10.23~31.75 kDa, and theoretical isoelectric points between 6.47~9.64 (Table 2).

### 2.3. Analyses of Gene Structures and Protein Motifs of EPSPS and BAR Genes

**Figure 2:**
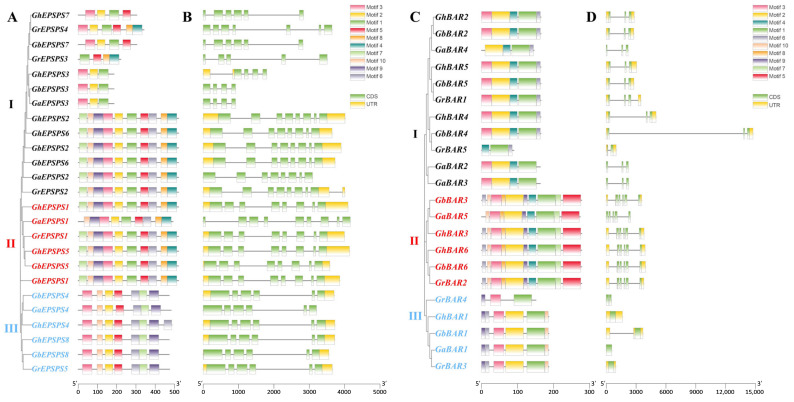
Conserved motif and gene structure analysis and phylogenetic tree construction of *EPSPS* and *BAR* family members. Conserved protein motifs of the *EPSPS* (**A**) and *BAR* (**C**) gene. Gene structure of the *EPSPS* (**B**) and *BAR* (**D**) gene. The conserved motifs in the *EPSPS* and *BAR* gene proteins are indicated by colored boxes. The green and yellow boxes represent CDS and UTR, respectively. The length of the boxes and lines are scaled according to the length of the gene.

The conserved motifs and gene structures of the 25 *EPSPS* family members were analyzed (Figure 2A,B), and the *EPSPS* family was divided into three subgroups (I, II, and III) based on Motif characteristics and gene structure. In subgroup I, most genes contained eight exons, and all but six genes had ten Motifs, while the rest had three to six Motifs. The majority of genes contained eight exons, with *GaEPSPS1* having the highest number of introns (nine) and the gene not having a Motif7, while the rest had ten Motifs in subgroup II. All genes had six exons and had seven identical Motifs in subgroup III.

The 22 *BAR* family members are divided into three subgroups (I, II, and III) (Figure 2C,D). Subgroup I has all genes with three exons except for *GrBAR5*, which contains only one exon, and the number of Motifs ranges from three to five; subgroup II family members all have five exons, except for *GaBAR5*, which does not have Motif6, and the rest of the genes have ten Motifs; subgroup III has the majority of genes containing two exons, four genes have six Motifs, and the number of Motifs ranges from three to six.

These results suggest that the cotton *EPSPS* and *BAR* gene families are evolutionarily well conserved, and some *EPSPS* and *BAR* members in the same subgroup have similar gene structures.

### 2.4. Phylogenetic Analysis of the EPSPS and BAR Gene Family

**Figure 3:**
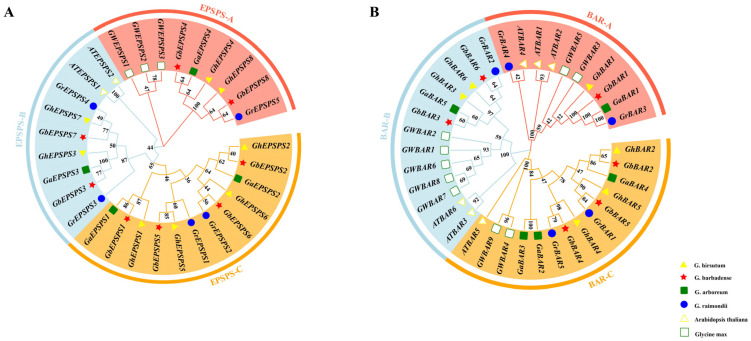
Phylogenic tree of the *EPSPS* and *BAR* family members. Phylogenetic tree of the *EPSPS* (**A**) and *BAR* (**B**) gene family of six species. By multiple sequence alignment of 30 *EPSPS* proteins and 37 *BAR* proteins from each of the six species, the *EPSPS* gene family was divided into three subgroups, EPSPS-A (9 proteins), EPSPS-B (9 proteins) and EPSPS-C (12 proteins); the *BAR* gene family was divided into three subgroups, BAR-A (10 proteins), BAR-B (13 proteins) and BAR-C (14 proteins) subgroups.

To reveal the evolutionary relationships of the *EPSPS* gene family, a phylogenetic tree was constructed using multiple sequence alignment analysis of the protein sequences of twenty-five cotton, two *Arabidopsis,* and three soybean *EPSPS* genes (Figure 3A). The results showed that the *EPSPS* gene family was divided into three subgroups (A, B, and C). Subgroup A includes two branches, with six cotton *EPSPS* family members as one branch and three soybean family members as one branch, indicating the relative evolutionary independence of *EPSPS* genes in plants. Subgroup B contains two *Arabidopsis* and seven cotton *EPSPS* family members, indicating that *EPSPS* is evolutionarily conserved and homologous. Subgroup C includes twelve members of the cotton *EPSPS* family. It was further found that only cotton *EPSPS* genes are found inside subgroup C, indicating that subgroup C is a unique *EPSPS* gene formed during the evolutionary history of cotton, suggesting that cotton *EPSPS* genes may have generated functional differentiation during evolution.

The phylogenetic tree showed the twenty-two *BAR* family members were divided into three subgroups (A, B, and C) along with six *Arabidopsis* and nine soybean *BAR* family members (Figure 3B). Among these subgroups, subgroup C contains the most members with eleven cotton *BAR* family members, subgroup B contains six cotton *BAR* family members, and subgroup A contains five cotton *BAR* family members. Subgroup A contains two *Arabidopsis* and three soybean genes, subgroup B contains two *Arabidopsis* and five soybean genes, and subgroup C has one *Arabidopsis* and two soybean genes, indicating that the cotton *BAR* family members are closely related to the *Arabidopsis* and soybean family members, and they may have conserved physiological and biochemical functions.

### 2.5. Collinearity Analysis of EPSPS and BAR Family Members

**Figure 4:**
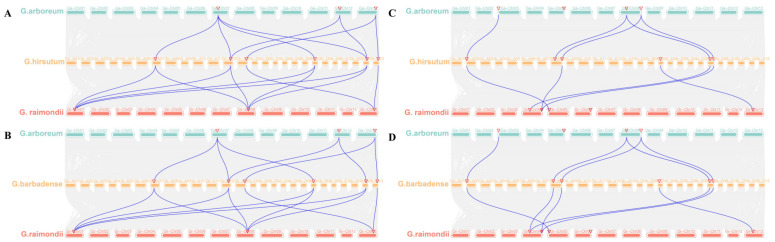
Collinearity analysis of *EPSPS* and *BAR* genes. (**A**) Collinearity analysis of *EPSPS* genes among *G. hirsutum*, *G. arboreum,* and *G. raimondii*. (**B**) Collinearity analysis of *EPSPS* genes among *G. barbadense*, *G. arboreum,* and *G. raimondii*. (**C**) Collinearity analysis of *BAR* genes among *G. hirsutum*, *G. arboreum,* and *G. raimondii*. (**D**) Collinearity analysis of *BAR* genes among *G. barbadense*, *G. arboreum,* and *G. raimondii*. The grey lines indicate colinear blocks, and the blue lines indicate the homozygous pairs of common *G. hirsutum* or *G. barbadense* with *G. arboreum* and *G. raimondii*, respectively. The red inverted triangle represents the location of the *EPSPS* and *BAR* genes.

Analysis of collinearity between tetraploid and diploid cotton showed that six of the *EPSPS* genes in each of *G. hirsutum* and *G. barbadense* were colinear with three *GaEPSPS* genes and equally colinear with three *GrEPSPS* genes (Figure 4A,B). In contrast, *GhEPSPS3* and *GhEPSPS7*, *GbEPSPS3* and *GbEPSPS7* have no co-linear genes with diploids, *GaEPSPS2*, *GrSEPSPS3*, and *GrSEPSPS4* have no co-linear relationships with tetraploid genes. Overall, there was a more conservative co-linearity between the cotton *EPSPS* family genes.

In *G. hirsutum*, 5 *GhBAR* genes were colinear with three *GaBAR* genes, and four *GhBAR* genes were colinear with six *GrBAR* genes (Figure 4C). In *G. barbadense*, five *GbBAR* genes were colinear with three *GaBAR* genes, and six *GbBAR* genes were colinear with four *GrBAR* genes (Figure 4D). While *GhBAR4* had no co-linearity with *GaBAR*, *GhBAR2* had no co-linearity with *GrBAR*, and *GaBAR2*, *GaBAR3*, and *GrBAR4* had no co-linearity with the tetraploid genome; the results indicated that *BAR* genes were more divergent between diploid and tetraploid cotton.

### 2.6. Analysis of cis-Elements in the Promoter of EPSPS, BAR Family Members

**Figure 5:**
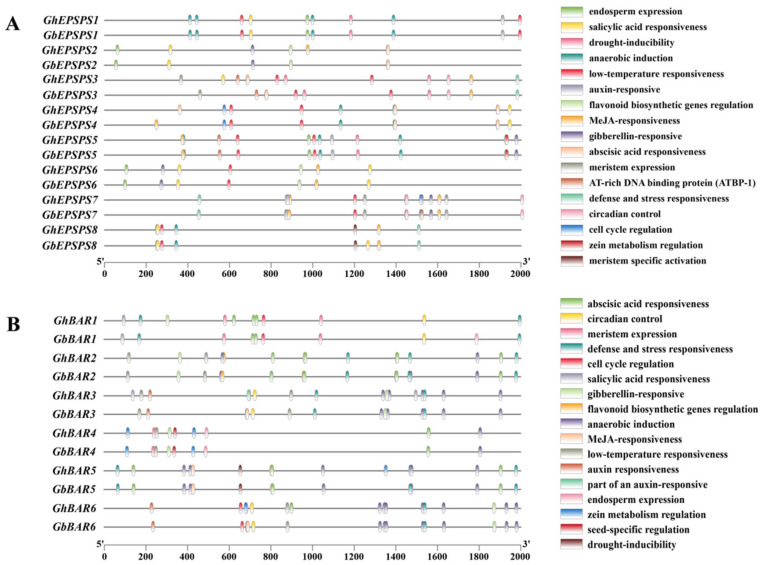
*cis*-element analysis of family members in tetraploid cotton. Members of the *EPSPS* (**A**) and *BAR* (**B**) gene family. Colored boxes indicate the different *cis*-elements in the promoters.

To preliminarily elucidate the possible regulatory mechanisms of the *EPSPS* family of genes in *G. hirsutum* and *G. barbadense*, the promoter (a 2000 bp DNA sequence upstream of ATG) was analyzed using the PlantCARE database (Figure 5A). The results show that each member of the *EPSPS* family contains a variable number of *cis*-elements. In addition to the conventional *cis*-elements, they can be divided into three categories: growth and development-related, stress-related, and hormone-related. Among them, endosperm expression (8), anaerobic induction and low-temperature response (22 each), and abscisic acid response (15) accounted for the largest number in each. Comparison of the *cis*-elements of *G. hirsutum* with those of *G. barbadense* revealed an increase in the *cis*-element of the MeJA reaction in *GhEPSPS2* compared with *GbEPSPS2* and in *GhEPSPS4* compared with *GbEPSPS4*. *GhEPSPS3* and *GhEPSPS8* have an additional *cis*-element for the salicylic acid reaction compared to *GbEPSPS3* and *GbEPSPS8*. The remaining homologous genes in both *G. hirsutum* and *G. barbadense* have the same types. The enrichment of the above response elements suggests that the cotton *EPSPS* genes may be involved in plant growth and development and in response to environmental stress.

The *cis*-elements of the *BAR* family gene promoters were analyzed between *G. hirsutum* and *G. barbadense* (Figure 5B). They were also divided into three categories: growth and development-related, stress-related, and hormone-related. Among them, anaerobic induction (41) and abscisic acid reaction (23) accounted for the largest number of each. Comparing the *cis*-elements of *G. hirsutum* with those of *G. barbadense*, we found that *GhBAR1* increased endosperm expression and gibberellin-related *cis*-elements compared to *GbBAR1*, *GhBAR3* increased low temperature and growth hormone partially related *cis*-elements compared to *GbBAR3*, *GhBAR5* increased maize alcoholic protein metabolism *cis*-elements compared to *GbBAR5*, and *GhBAR6* increased the *cis*-elements of maize alcoholic protein metabolism and abscisic acid compared to *GbBAR6*. This suggests that members of the *BAR* family have the potential to play important roles in the above pathways.

### 2.7. Expression Analysis of GhEPSPS, GhBAR Family Members

The expression patterns of *GhEPSPS* and *GhBAR* family members were analyzed in eight tissues of cotton (root, stem, leaf, pistil, stamen, calyx, petal, and receptacle) and four types of stresses (low temperature, high temperature, salt, and drought) based on published transcriptome data of *G. hirsutum* TM-1 genome.

**Figure 6:**
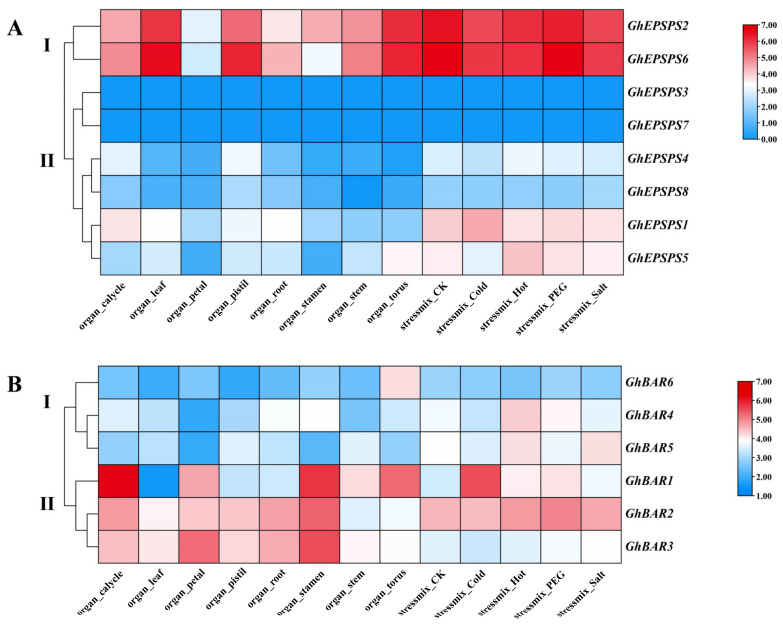
Expression levels of *GhEPSPS* and *GhBAR* in cotton under different tissues and stresses. (**A**) Expression levels of *GhEPSPS* genes. (**B**) Expression levels of *GhBAR* genes. Colors indicate gene expression levels. Red and blue colors indicate high and low expression levels, respectively.

The members of the *GhEPSPS* family were divided into two expression patterns (Figure 6A). Pattern I contained two *GhEPSPS* members, which were expressed in various tissues and were highly expressed in leaves, pistils, and receptacles. Pattern II included six *GhEPSPS* members, with *GhEPSPS1* being moderately highly expressed in the calyx and the remaining *GhEPSPS* genes being lowly expressed in all tissues. These results indicated that *GhEPSPS* genes play a role in different tissues of cotton, with *GhEPSPS2* and *GhEPSPS6* exhibiting significant tissue-specific expression. Most *GhEPSPS* genes showed no significant change in expression levels after abiotic stress treatment. Only a portion of *GhEPSPS* gene expression was induced by low-temperature treatment, such as the upregulation of *GhEPSPS1* and *GhEPSPS4* expression, indicating that these genes are involved in response to low-temperature stress.

*GhBAR* family members are also divided into two expression patterns (Figure 6B). In pattern I, *GhBAR6* was highly expressed in the flower receptacle, while the remaining *GhBAR* genes were less expressed in various tissues. In pattern II, the *GhBAR1* gene was more expressed in the calyx, stamens, and receptacle, while the other two genes were highly expressed in tissues other than the stem and receptacle. Therefore, the *GhBAR* family exhibits tissue-specific expression. Under different stress, *GhBAR1* was highly expressed under low temperature and drought stress, *GhBAR4* responded to high-temperature stress, and *GhBAR5* responded to both high temperature and drought stress.

### 2.8. Expression Levels of EPSPS and BAR Family Members after Herbicide Spraying by qRT-PCR

**Figure 7:**
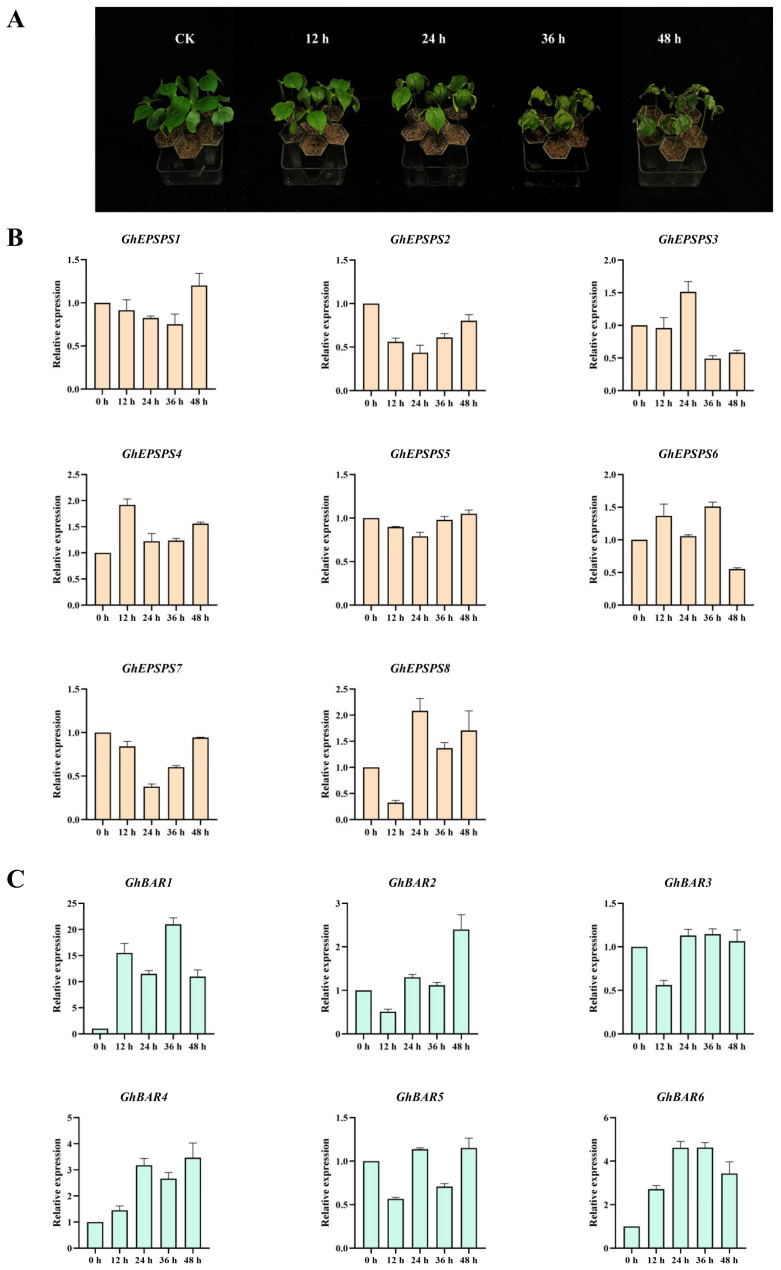
Expression patterns of *EPSPS* and *BAR* genes in true leaves under herbicide stress. (**A**) Nongda 601 seedlings at 0 h, 12 h, 24 h, 36 h, and 48 h. The expression levels of *EPSPS* (**B**) and *BAR* (**C**) genes at 0 h, 12 h, 24 h, 36 h, and 48 h after herbicide treatment, respectively. The data were shown as mean ± standard error for three biological replicates.

To investigate whether the *EPSPS* and *BAR* families respond to herbicide stress, cotton leaves were treated with herbicide spraying. It was found that the cotyledons of cotton began to show mild wilting at 24 h after herbicide treatment. At 36 h, the cotyledons and true leaves showed a wilting and shrinking state, and the leaf shrinkage became more pronounced at 48 h (Figure 7A). We further detected the expression levels of 14 genes in the *GhEPSPS* and *GhBAR* families at different stages after herbicide treatment by qRT-PCR. The results showed that genes were generally upregulated. *GhEPSPS3* was upregulated by about 10 times at 24 h, while *GhEPSPS4* and *GhEPSPS5* showed an upregulation trend with about 0.5 times compared to that of the control. *GhEPSPS1* and *GhEPSPS8* showed a significant upregulation trend at 36 h, while *GhEPSPS2* showed an upregulation trend at 48 h, which was about twice as high as before. *GhEPSPS3* had the highest expression level after herbicide stress (Figure 7B). Most of the *GhBAR* genes are upregulated, with *GhBAR1*, *GhBAR4*, and *GhBAR6* showing the most significant upregulation at 36 h. Among them, *GhBAR1* is particularly upregulated, with an expression level of about 12 times that of untreated at 12 h and over 18 times that of untreated at 36 h. In addition, *GhBAR1* has the highest expression level in the *BAR* family under herbicide stress. *GhBAR4* and *GhBAR5* showed a significant upregulation trend at 48 h (Figure 7C). This indicated that *GhEPSPS* and *GhBAR* families were induced to express by herbicides and may play an important role in the resistance to herbicides.

## 3. Discussion

Allotetraploid cotton (AADD) originated from the hybridization of A genome species as the maternal parent and D genome species as the pollen donor. The A genome species originated from *G. arboreum* and *G. herbaceum* (A2), while the D genome species originated from *G. raimondii* [40,41]. Two diploid ancestral species have undergone over a period of one to two million years of heteropolyploidization to form tetraploid cultivated species [42]. The number of genes in tetraploid *G. hirsutum* and *G. barbadense* should be twice or more than that in diploid *G. arboreum* and *G. raimondii* [43]. We identified eight *EPSPS* genes in *G. hirsutum* and *G. barbadense*, with consistent distribution on chromosomes, with four genes in the A subgenome and four genes in the D subgenome. It was quantitatively similar to the *EPSPS* genes identified in two diploid cotton varieties, indicating that the *EPSPS* genes of allotetraploid cotton have not undergone separate replication or significant gene loss events during evolution [44]. However, for the *BAR* gene family, there are five genes in diploid and six genes in tetraploid cotton. It is speculated that it may be the result of the elimination of some genes in the *BAR* family during the formation of the tetraploid genome.

The expression of the *EPSPS* gene may be different in tissues and organs [45]. Under the stresses, plants regulate the expression of genes in order to resist the harm of adversity and ensure the normal physiological function of plant cells and the growth and development of plants [46]. In rice, *LOCOs06g04280* is dominantly expressed in the root. In *Arabidopsis*, the expression of *AT1G48860* in leaves was extremely significant. In tobacco, the accumulation of *EPSPS* transcripts was highest in mature leaves. After 14 days of herbicide stress, the expression of *NtEPSPS* was significantly upregulated, which was more than twice that of the control [21]. The expression of the *IbEPSPS* gene cloned from sweet potato was the highest in the stem. After spraying herbicide on isolated shoots, *IbEPSPS* gene expression decreased and then increased [47]. The *EPSPS* gene of *G. hirsutum* (Coker312) is predominantly expressed in the true leaves [48]. The expression level of *EPSPS* increased after glyphosate stress in cotton strain Y18 [37]. The expression of the *CaEPSPS* gene in field bindweed significantly increased under glyphosate treatment. Introducing the *CaEPSPS* gene into *Arabidopsis* revealed stronger glyphosate tolerance [49]. In this study, we found that *GhEPSPS2* and *GhEPSPS6* were also predominantly expressed in leaves, and herbicide treatment could increase the expression of *EPSPS*, indicating that *EPSPS* family genes could respond to herbicide stress. *EPSPS* is the only glyphosate target enzyme in plants, and its expression level can directly affect the resistance of plants to glyphosate. Plants improve their tolerance to glyphosate by increasing *EPSPS* gene expression to ensure normal plant life activities, which is a common glyphosate resistance mechanism at present, but the specific response mechanism still needs to be further explored.

*EPSP* synthases can be divided into two types, EPSPS I and EPSPS II, according to the principle of whether antigen and antibody cross-react and whether amino acid sequence homology is less than 50% [50]. The natural *EPSPS* gene of plants is EPSPS I and is not resistant to herbicides. This type of *EPSPS* gene is sensitive to glyphosate, and after gene mutation, it is possible for organisms to acquire tolerance to glyphosate [51]. In addition, Monsanto transferred *EPSP* synthase derived from *Agrobacterium* sp. CP4 into plants to develop genetically modified crops resistant to glyphosate [23]. This *EPSPS* genes isolated from *Agrobacterium tumefaciens* are EPSPS II, which tend to show higher catalytic efficiency and are not inhibited by glyphosate in the presence of glyphosate [52]. At present, most of the transgenic crops resistant to glyphosate are created using EPSPS II [53]. *Arabidopsis* has two natural *EPSPS* loci, *AtEPSPS1*(*AT1G48860*) and *AtEPSPS2*(*AT2G45300*), which are highly expressed throughout development [54]. It was found that overexpression of the natural gene encoding 5-enolpyruvate oxalate synthase (EPSP) may increase the fertility of *Arabidopsis* [22]. Therefore, we speculated that the natural *EPSPS* genes may have the same regulatory mechanism in cotton, and overexpression of *EPSPS* genes may improve the reproductive ability of cotton, laying a foundation for further improving cotton. Among them, *AtEPSPS2*(*AT2G45300*) is the upregulated expression gene of Ca^2+^ response, and Ca^2+^ transient mediates the response to environmental stresses, including salt, drought, cold, heat, ultraviolet, etc., which is the key to plant resistance to biological and abiotic stresses [55]. This gene is homologous to the cotton *EPSPS* gene and may play a role in ion regulation. In cotton, *GhEPSPS1* and *GhEPSPS4* are upregulated under low-temperature treatment, which may be attributed to Ca^2+^ involvement in the response process of cotton to low-temperature stress. In plants, *cis*-regulatory elements are associated with ABA in Ca^2+^ response gene promoters. Among the hormone-related *cis*-acting elements in the *EPSPS* promoter response of tetraploid cotton, the homeopathic elements of abscisic acid reaction accounted for the largest number. Therefore, we hypothesized that *EPSPS* genes in cotton activate in vivo gene expression through Ca^2+^ response to abscisic acid *cis*-acting elements.

The *BAR* gene has been introduced into many plant species as an optional marker during transformation and provides tolerance to glufosinate [56]. Plants with the *BAR* gene can survive under glufosinate treatment, providing an excellent system for screening transgenic plants through a single treatment [27]. At present, the *BAR* gene was isolated from *Streptomyces hygroscopicus* in existing transgenic crops resistant to phosphine glufosinate, and it was transferred into the crops to make them resistant to phosphine glufosinate. At present, researchers have studied different crops with *BAR* gene transfer [30,57,58,59,60]. *BAR* gene not only can improve resistance to the herbicide glufosinate but also play a role in the salt stress and flowering of plants. In *Arabidopsis thaliana*, *AtBAR1* (*AT2G32020*) was upregulated under salt stress [61]. In order to adapt to the salt-stressed environment, plants achieve salt tolerance by activating the SOS signaling pathway [62], which requires the combined activity of three proteins to prevent the accumulation of Na^+^ so as to achieve plant salt tolerance [63]. *AtBAR1* is also involved in the regulation of aging, oxidative stress, defense, and plant hormones [64]. In this study, the up-regulation trend of *GhBAR1* was the largest after herbicide treatment, and the expression level was about 6~18 times that in the untreated condition, which was much higher than that of other family members. Phylogenetic analysis showed that *AtBAR1* and *GhBAR1* belong to the same clade, indicating that they are closely related and may have similar functions. Promoter *cis*-elements analysis showed that *GhBAR1* responded to cell cycle regulation, defense and stress response, salicylic acid, and abscisic acid. Transcriptome data also showed that *GhBAR1* was upregulated under salt stress. At the same time, *GhBAR1* had a broad response under cold stress, which could be a potential gene for the study of herbicide resistance mechanisms and their function under abiotic stress. *AtBAR3*(*AT3G02980*) is involved in the development of leaves and flowers as well as male and female gametes. Overexpression results in narrower, longer rosette leaves, faster stem elongation, and earlier flowering [65]. *GhBAR1* is highly expressed in stamens, and *GhBAR2* and *GhBAR3* are moderately and highly expressed in the leaves of stamens and pistils. These genes may play a role in the reproductive organs and fiber of cotton.

## 4. Materials and Methods

### 4.1. Identification of EPSPS and BAR Family Members

The genome sequence and annotation data of *Gossypium hirsutum* (HEBAU), *G. barbadense* (HEBAU), *G. arboreum* (CRI), and *G. raimondii* (JGI) [39,66,67] were downloaded from CottonFGD (http://www.cottonfgd.org/, accessed on 27 November 2022). The Hidden Markov Model (HMM) database (Protein families database of alignments and HMM, Pfam database) website (http://pfam.xfam.org/, accessed on 27 November 2022) was used to download the conserved structural domains EPSP synthase (3-phosphoshikimate 1-carboxyvinyltransferase) (EPSP_synthase, PF00275) for the *EPSPS* gene and Acetyltransferase (GNAT) domain (Acetyltransf_4, PF13420) for the *BAR* gene. The *EPSPS* and *BAR* genes of four cotton species were obtained using the Simple HMM search function in TBtools software [68], and then the *EPSPS* and *BAR* family member sequences were obtained by using the NCBI CD-Search website (https://www.ncbi.nlm.nih.gov/Structure/cdd/wrpsb.cgi, accessed on 27 November 2022) to remove incomplete sequences of conserved structural domains.

### 4.2. Characterization of EPSPS and BAR Family Members

The protein and transcriptional features of the obtained *EPSPS* and *BAR* family members were extracted and summarized by the Date Fetch and Enrichment function of CottonFGD (https://cottonfgd.net/analyze/, accessed on 27 November 2022). The chromosomal location information of the resulting family members was visualized and constructed using the Gene Location Visualize from GTF/GFF function in TBtools software (V2.001), respectively.

### 4.3. Conserved Domain and Gene Structure Analysis of EPSPS and BAR Family Members

The amino acid sequences of the identified family members were extracted by TBtools software, and the motifs of the *EPSPS* and *BAR* family members were further analyzed using MEME (http://meme-suite.org/tools/meme, accessed on 17 December 2022), where the number of motifs was set to a value of 10 and the rest by default. The structure of the family members was analyzed using the Visualize Gene Structure function in the TBtools software.

### 4.4. Phylogenetic Relationship of EPSPS and BAR Family Members

Family member gene sequences for four cotton species were obtained from CottonFGD (https://cottonfgd.net/, accessed on 27 November 2022), Arabidopsis family member gene sequences from TAIR (https://www.arabidopsis.org/, accessed on 10 December 2022), and soybean family member sequence genes from MBKBASE (http://www.mbkbase.org/, accessed on 10 December 2022). The obtained *EPSPS* and *BAR* family member sequences were compared using MEGA-X software [69], and the phylogenetic tree was constructed using Neighbor-Joining (NJ), with the Bootstrap value set to 1000 and the rest as default values.

### 4.5. Analysis of cis-Elements of Promoters of EPSPS and BAR Family Members

DNA sequence information of 2000 bp upstream of the two tetraploid cotton family members was extracted by the Date Fetch and Enrichment function of CottonFGD (https://cottonfgd.net/analyze/, accessed on 22 December 2022). Possible *cis*-elements were analyzed and collated using the PlantCARE (http://bioinformatics.psb.ugent.be/webtools/plantcare/html/, accessed on 22 December 2022) website. Visualization was achieved through the Simple BioSequence Viewer function in TBtools.

### 4.6. Collinearity Analysis of EPSPS and BAR Family Members

The genomes of diploid and tetraploid cotton were analyzed by the One Step MCScanX—Super Fast function in TBtools software, followed by extraction and visualization of the covariance of *EPSPS* and *BAR* members.

### 4.7. Expression Analysis of GhEPSPS and GhBAR Family Members in Different Tissues and under Biotic and Abiotic Stresses

Download transcriptome data from the NCBI Sequence Read Archive database for eight tissues (root, stem, leaf, pistil, stamen, calyx, petal, and receptacle) and four stresses (cold, heat, drought, and salt stress) of *G. hirsutum* ‘TM-1′(PRJNA490626) [70]. The transcriptomic data were log_2_ (1+FPKM) normalized. HeatMap functional software in TBtools was used to map the expression of *EPSPS* and *BAR* family members.

### 4.8. Gene Expression Analysis of EPSPS and BAR Family Genes in Different Periods after Herbicide Stress

The *G. hirsutum* variety Nongda 601 was grown in a greenhouse culture, and the leaves were uniformly sprayed with 8 mL/L^−1^ of herbicide for up to 30 days. RNA was extracted from the leaves before (0 h) and 12, 24, 36, and 48 h after spraying using FastPureRPlant Total RNA Isolation Kit (purchased from Vazyme, Nanjing, China), respectively. The obtained RNA was reverse transcribed into cDNA using the HiScript III RT SuperMix For qPCR (+gDNA wiper) reverse transcription kit (purchased from Vazyme), and RT-PCR reaction was performed using Taq Pro Universal SYBR qPCR Master Mix (purchased from Vazyme) on Roche LightCycler 96 (Roche, Basel, Switzerland). The program settings refer to the instructions. The used primers were shown in Table 3, and the expression of GhUBQ14 was used as an internal reference. Relative expression was calculated using 2^−△△CT^ and three biological replicates were set.

## 5. Conclusions

In summary, this study is the first report on the genome-wide characteristics of *EPSPS* and *BAR* gene families in diploid and tetraploid cotton. We identified 25 *EPSPS* genes and 22 *BAR* genes in the cotton genomes. The number of *EPSPS* genes in tetraploid cotton is twice that in diploid cotton, while the number of *BAR* genes in tetraploid and diploid cotton is almost the same. The expression levels of *GhEPSPS* are significantly higher in leaves and pistils, while most *GhBAR* genes are highly expressed in calycle and stamen. Among the *EPSPS* and *BAR* genes, the expression levels of *GhEPSPS3*, *GhEPSPS4,* and *GhBAR1* were significantly upregulated under herbicide treatment. This study provides a systematic and profound understanding of the *EPSPS* and *BAR* families and contributes to further study on the function of *EPSPS* and *BAR* families under herbicide stress in cotton.

## Figures and Tables

**Table 1:** *EPSPS* family member information.

Gene	Gene ID	Chromosome	All Length (bp)	CDS Length (bp)	Number of Exons	Protein Length (aa)	Molecular Weight (kDa)	Isoelectric Point
*GhEPSPS1*	*GhM_A07G1799*	A07	4095	1566	8	521	55.49	8.08
*GhEPSPS2*	*GhM_A12G1349*	A12	4011	1566	8	521	55.59	7.83
*GhEPSPS3*	*GhM_A12G1350*	A12	1797	558	5	185	20.56	6.49
*GhEPSPS4*	*GhM_A13G2002*	A13	3723	1461	6	486	51.93	8.43
*GhEPSPS5*	*GhM_D07G1761*	D07	4137	1566	8	521	55.37	7.92
*GhEPSPS6*	*GhM_D12G1287*	D12	3643	1566	8	521	55.6	8.38
*GhEPSPS7*	*GhM_D12G1288*	D12	2836	915	6	304	32.76	6.24
*GhEPSPS8*	*GhM_D13G1901*	D13	3719	1419	6	472	50.45	8.56
*GbEPSPS1*	*GbM_A07G1760*	A07	3863	1566	8	521	55.48	8.26
*GbEPSPS2*	*GbM_A12G1269*	A12	3900	1566	8	521	55.6	7.83
*GbEPSPS3*	*GbM_A12G1270*	A12	915	558	4	185	20.56	5.67
*GbEPSPS4*	*GbM_A13G1999*	A13	3703	1419	6	472	50.23	8.14
*GbEPSPS5*	*GbM_D07G1761*	D07	3580	1566	8	521	55.45	7.92
*GbEPSPS6*	*GbM_D12G1236*	D12	3730	1566	8	521	55.6	8.38
*GbEPSPS7*	*GbM_D12G1237*	D12	2820	915	6	304	32.76	5.96
*GbEPSPS8*	*GbM_D13G1869*	D13	3554	1419	6	472	50.39	8.56
*GaEPSPS1*	*Ga07G1630*	Chr07	4159	1479	9	492	52.68	7.2
*GaEPSPS2*	*Ga12G1903*	Chr12	3091	1566	8	521	55.54	7.72
*GaEPSPS3*	*Ga12G1902*	Chr12	915	558	4	185	20.62	5.68
*GaEPSPS4*	*Ga13G1869*	Chr13	3205	1449	6	482	51.46	8.3
*GrEPSPS1*	*Gorai.001G174400*	Chr01	3994	1566	8	521	55.32	7.85
*GrEPSPS2*	*Gorai.008G113600*	Chr08	4053	1566	9	521	55.52	8.73
*GrEPSPS3*	*Gorai.008G113700*	Chr08	3508	666	5	221	24.12	6.87
*GrEPSPS4*	*Gorai.008G113800*	Chr08	3642	1026	7	341	37.52	7.73
*GrEPSPS5*	*Gorai.013G173300*	Chr13	3731	1425	6	474	50.69	8.62

**Table 2:** *BAR* family member information.

Gene	Gene ID	Chromosome	All Length (bp)	CDS Length (bp)	Number of Exons	Protein Length (aa)	Molecular Weight (kDa)	Isoelectric Point
*GhBAR1*	*GhM_A02G0363*	A02	1627	561	1	186	21.23	6.47
*GhBAR2*	*GhM_A08G1199*	A08	2810	495	3	164	18.4	8.43
*GhBAR3*	*GhM_A08G2862*	A08	3804	834	6	277	31.75	9.03
*GhBAR4*	*GhM_D03G0774*	D03	5007	495	3	164	18.51	8.43
*GhBAR5*	*GhM_D08G1150*	D08	3053	495	3	164	18.43	8.99
*GhBAR6*	*GhM_D08G2805*	D08	3909	834	6	277	31.72	9.04
*GbBAR1*	*GbM_A02G0373*	A02	3684	561	2	186	21.23	6.47
*GbBAR2*	*GbM_A08G1080*	A08	2763	495	3	164	18.4	8.43
*GbBAR3*	*GbM_A08G2815*	A08	3570	834	6	277	31.73	9.18
*GbBAR4*	*GbM_D03G0750*	D03	14,746	495	3	164	18.51	8.43
*GbBAR5*	*GbM_D08G1153*	D08	2778	495	3	164	18.43	8.99
*GbBAR6*	*GbM_D08G2789*	D08	3941	834	6	277	31.72	9.04
*GaBAR1*	*Ga03G0387*	Chr03	561	561	1	186	21.23	6.52
*GaBAR2*	*Ga05G4007*	Chr05	2224	489	3	162	18.37	9.51
*GaBAR3*	*Ga05G4011*	Chr05	2250	489	3	162	18.35	9.37
*GaBAR4*	*Ga08G1028*	Chr08	2202	438	3	145	16.43	8.82
*GaBAR5*	*Ga08G2611*	Chr08	2415	822	5	273	31.01	8.77
*GrBAR1*	*Gorai.004G110900*	Chr04	3619	495	4	164	18.43	9.05
*GrBAR2*	*Gorai.004G256900*	Chr04	3777	834	6	277	31.75	9.08
*GrBAR3*	*Gorai.005G044300*	Chr05	957	561	1	186	21.17	5.85
*GrBAR4*	*Gorai.006G233400*	Chr06	508	453	2	150	17.23	9.64
*GrBAR5*	*Gorai.013G110600*	Chr13	992	270	2	89	10.23	8.22

**Table 3:** Primer information for qRT-PCR.

Primers	Sequence (5′–3′)	Primers	Sequence (5′–3′)
GhEPSPS1-qF	GAAATCCCTCTGGAAGGAAACA	GhBAR1-qF	GAACAAGGTTGTGCCTCACCCT
GhEPSPS1-qR	GCAGTAGGACCATCAGCA	GhBAR1-qR	TGCCTGAATTTGCACTCACCGA
GhEPSPS2-qF	TGATGGGTGCCAAAGTCACCTG	GhBAR2-qF	AAGATGCCATCAACTTCTATC
GhEPSPS2-qR	AAGAGTCATAGCAACGTCCGGC	GhBAR2-qR	GTAAGAACAAAGCAGTCGGGAG
GhEPSPS3-qF	ACGAGCCGTCCTCAAAGGTTAC	GhBAR3-qF	CAAAGATGTCGTTCAATTGCG
GhEPSPS3-qR	CATATTGCGGTTCCAACATTCC	GhBAR3-qR	GACCCCGGGTTTCGTTACC
GhEPSPS4-qF	AGGAGTCCGTGTTTGACAACCG	GhBAR4-qF	CGGGTTTGAAATCACCGAGACA
GhEPSPS4-qR	TAGTGCATTGCTCCCGCTAACC	GhBAR4-qR	TTTGTTCGCTTGAGATGTAGTG
GhEPSPS5-qF	CCGGACCACCAAGAAATCCCTC	GhBAR5-qF	CCATCCGTGTGTACATCATGACA
GhEPSPS5-qR	CTGCTGTCACATTTGGTTCGCC	GhBAR5-qR	TAGAAGTTGATGGCATCTTCA
GhEPSPS6-qF	ATCACGGGTGGGACTGTCACG	GhBAR6-qF	GCTTACGCGTTCGATGCAGGTA
GhEPSPS6-qR	CCGCATCCTTCTACCGTGACAG	GhBAR6-qR	ATCGAGTTCGGATCGAGGCAAG
GhEPSPS7-qF	ATAAACGGAAAGGGTGGTCTTC	UBQ14-F	CAACGCTCCATCTTGTCCTT
GhEPSPS7-qR	AGCTAAATGAGCTGCCATGAGT	UBQ14-R	TGATCGTCTTTCCCGTAAGC
GhEPSPS8-qF	CTTCTCCACAACCTTCCCAATG		
GhEPSPS8-qR	AATCAACCTCCACTTTGCCAACC		

## Data Availability

The data presented in this study are available within the article.
